# Making connections across silos: intimate partner violence, mental health, and substance use

**DOI:** 10.1186/s12905-017-0372-4

**Published:** 2017-04-12

**Authors:** Robin Mason, Marni Wolf, Susan O’Rinn, Gabrielle Ene

**Affiliations:** 1 Women’s College Research Institute, Women’s College Hospital, 76 Grenville Street, Toronto, ON M5S 1B2 Canada; 2 Dalla Lana School of Public Health and Department of Psychiatry, University of Toronto, 155 College Street, Toronto, ON M5T 3 M7 Canada

**Keywords:** Violence against women, Intimate partner violence, Domestic violence, Mental health, Substance abuse, Addictions, Education, Training, Inter-professional education

## Abstract

**Background:**

Untold numbers of women worldwide are survivors of intimate partner violence (IPV) with a substantial number of these experiencing co-occurring mental health and substance use problems. Despite the complex interconnections among these problems, funding mechanisms and organizational structures and mandates have been designed to address just a single, focal problem. One of the challenges for frontline providers is the lack of effective, evidence-informed inter-professional education or training to help them identify and appropriately respond to co-occurring problems. We developed an evidence-informed, competency-based curriculum to address this gap. In this paper we report on its effectiveness in increasing knowledge, changing beliefs and enhancing skills of frontline workers from all three sectors.

**Methods:**

The curriculum consists of multiple elements: a text manual; an interactive, online series of modules; and, an in-person workshop. Frontline workers (*n* = 1111) in the violence against women (VAW) (*n* = 499), mental health (*n* = 229), addiction treatment (*n* = 167), and associated sectors (*n* = 149) were recruited to attend the workshop and instructed to read the manual or complete the online modules before attending. Some failed to respond (*n* = 67). Online pre- and post-tests were used to assess changes in knowledge, beliefs and skills; evaluations of the workshop were also collected.

**Results:**

Matched pre- and post-tests were available for over half of the participants (*n* = 624). Results show statistically significant improvements across all six competency domains from pre to post-test (*p* <0.0001). Significant changes in participants’ knowledge and stigmatizing beliefs were achieved. There was no correlation among differences in sector, age, size of organization, years of experience or prior training. Participant feedback made evident prior misconceptions about women experiencing co-occurring problems, improved understanding about the need to bridge silos, as well as the need for enhanced self-care.

**Conclusions:**

An educational intervention designed to sensitize frontline workers to the realities of women’s experiences of co-occurring problems, educate about the challenges of accessing help when there are co-occurring problems, and bridge discipline and practice-based silos, can effectively challenge and alter providers’ negative attitudes and stigmatizing beliefs. Decreasing stigmatizing beliefs and increasing knowledge has the potential to help survivors access needed help.

**Electronic supplementary material:**

The online version of this article (doi:10.1186/s12905-017-0372-4) contains supplementary material, which is available to authorized users.

## Background

### Intimate partner violence & Co-occurring issues

Intimate partner violence (IPV), also known as domestic violence (DV), is the most common form of violence experienced by women [1]. Women in every country, of every age, background, class, religion, and ability are victimized and their health and well-being affected. IPV “refers to any behavior within an intimate relationship that causes physical, psychological or sexual harm to those in the relationship” [1]. Lifetime prevalence rates suggest that 30% of ever-partnered women experience physical and/or sexual violence at the hands of an intimate partner [2]. Rates of IPV are highest among women, particularly younger women and those in dating relationships [3]. While some men experience partner violence, women are three times more likely to report being beaten, choked, sexually assaulted, or threatened with a gun or knife by their partner or ex-partner [4].

The impacts of IPV on women’s health are well-documented and include physical (e.g. chronic pain, injury, gastrointestinal, gynecological) [5], psychological (e.g. depression, PTSD, anxiety) and substance abuse challenges [6, 7]. “The evidence is irrefutable – women’s experiences of domestic violence are connected in complex and reciprocal ways with poor mental health and substance use problems” [8]. Despite the prevalence and interconnections among these co-occurring problems, policies and funding formulas have resulted in services designed to address a single problem and women having to identify a priority treatment issue [9]. Reflecting the sole focus of services, the education system has continued to deliver professional training programs that inadequately prepare graduates to address the complex, co-occurring problems some women experience [10]. The result is that many VAW, mental health and addiction treatment staff lack the necessary knowledge and skills to help women with co-occurring problems [11, 12].

The practice gap was recognized in the U.S. which launched the Substance Abuse and Mental Health Services Administration (SAMHSA) initiative in 1992 to make substance use and mental disorder information, services, and research more accessible [13]. SAMHSA funded the Women and Co-occurring Disorders and Violence Study to implement and evaluate outcomes from integrated services. Fourteen sites implemented integrated programs; nine evaluated program outcomes. Improvements from baseline to six months were shown for participants in both the treatment-as-usual and intervention groups although greater improvements in post-traumatic symptom severity and drug problem severity were shown for those receiving integrated care. However, findings were complicated by the differences in which integrated care was delivered across the various sites [14]. The training gap was also recognized [15], but a scoping review of training for the co-occurring problems revealed that specific content for proposed education or training was lacking [16]. We found no comparable Canadian initiatives.

### Bridging silos through education

To address the gap in education and training for frontline providers working in each of the three sectors (VAW, mental health, addiction treatment), we developed an evidence-informed, competency-based, cross-sectoral curriculum and training initiative. After completing a literature review of recommended practices for addressing the co-occurring problems and conducting 14 focus groups to learn about existing practices and current challenges in service delivery, we convened an expert advisory committee with members from the three sectors and women with lived experience of the co-occurring problems, to help define the core competencies staff should possess to provide appropriate care to women with complex needs.

Medical education has increasingly emphasized competency development as a pedagogical means to shift from rote learning to a focus on the achievement of specific outcomes, abilities and learner-centred approaches to teaching [17]. Drawing upon this educational trend, we distilled findings from the literature, focus groups, and expert advisory committee to ten core competencies that anyone working in any one of the three sectors should possess. After circulating these to external stakeholders, the competencies formed the basis of the multi-modal educational initiative that we called, “Making Connections: When Domestic Violence, Mental Health and Substance Use Co-occur”.

By combining didactic teaching with active learning methods [18] and including a text-based manual; an interactive, online learning system; a day-long, cross-sectoral training workshop; and, an online discussion room, we hoped to bridge the artificial silos governing practice. The Making Connections manual emphasizes the strengths and limitations of each of the three sectors’ differing frameworks, philosophies and practices and includes introductory modules on IPV, mental health and addictions; how these problems develop, create vulnerabilities and ultimately reinforce other conditions; what to do for clients experiencing these problems; how to respond to crisis situations; ways of collaborating across disciplines and sectors; and, self-care and compassion fatigue [19]. The online component of the curriculum includes content similar to the manual but introduces it in a highly interactive and visual way complete with quizzes, photos, illustrations and videos [20]. The workshop, a key element of training, brought together individuals from the same geographic area and each of the three sectors to brainstorm strategies for minimizing the negative effects of care silos; and, share expertise and experiences in supporting women with complex needs. Each workshop ended with a series of exercises focused on self-care.

Together these elements were designed to:

1. Increase participants’ knowledge about the ways in which IPV, mental health, and substance use intersect;
2. Change stigmatizing beliefs participants may have about women who experience these co-occurring problems;
3. Provide participants with the skills to support women who experience these problems.

## Methods

This mixed methods study used online pre- post-test surveys and an in-person evaluation with both closed and open-ended questions to assess changes in participants’ knowledge, beliefs and skills.

### Sample, participants and setting

Between June 2012 and June 2015, 52 workshops were held across the province of Ontario, Canada, in locations ranging from rural remote to urban. Each workshop could accommodate 25 participants recruited from the three designated sectors (12 participants from VAW shelters and counselling organizations, 6 from mental health agencies and 6 from substance use treatment settings). In some settings this ‘formula’ could not be achieved; for example, specialists in the treatment of addictions were not always locally available. When this occurred, individuals from other sectors (for example, child welfare, and justice) were invited to participate. All participants were required to complete the first three modules of the online program or text manual before attending the workshop. The remaining modules or chapters were completed after the workshop. The manual, online program and workshop were made available free of charge. To reduce the possibility of’no-shows’ at the workshop, participants were required to obtain their manager’s signature as part of registration. More than 1,100 individuals attended a workshop over the course of 3 years.

Members of the expert advisory committee were invited to participate in a one day training if they were interested in becoming workshop facilitators. A detailed facilitator manual was also developed and provided. This group of core facilitators had expertise in one of the three designated sectors (VAW, mental health, or addictions) and delivered the workshop with a local co-facilitator from a different sector. This helped ensure that the workshop was cross-sectoral, participants could recognize themselves in the materials presented, and that locally relevant issues were considered. To address project sustainability, local co-facilitators were then invited to participate in a second one day training to become a core facilitator.

### Data collection and statistical analysis

Participants completed a demographic form and pre-test (see Additional file 1) with workshop registration. Approximately three months after the workshop, participants were emailed the post-test. Once the post-test was received, a certificate of completion was emailed to the participant.

The pre/post-tests assessed participants’ knowledge, beliefs and self-rated competency. Knowledge and belief questions were coded as correct/incorrect; competency was rated on a Likert scale of 0 (not at all competent) to 5 (highly competent). Matched pre/post-test responses on knowledge and beliefs were analyzed using McNemar’s Test. For the total average score, a paired *t*-test was used. Unanswered questions from the knowledge and belief questions were considered incorrect.

Following the analysis of all matched pre- and post-tests, a median and interquartile range (IQR) was determined for each question related to self-rated skill/competency. Due to the asymmetric distribution of the data, the median and IQR are reported. The medians are compared by the Wilcoxon signed-rank test conducted for each question. Differing sample sizes are the result of unanswered questions in either the pre-test or post-test. Unanswered self-rated skill/competency questions were not included in the analyses.

In addition, at the end of each workshop participants completed an evaluation with both closed and open-ended questions. Participants rated the usefulness of each workshop activity (not at all, somewhat, a lot, very) while open-ended questions allowed them to share the most important knowledge attained and any intended changes to their own practice. A thematic analysis of the open-ended questions was completed by first reviewing a random sample of responses from different workshops. From this review, draft codes were developed. Coding of the open-ended responses was completed according to this template with the understanding that new codes would continue to be developed until no new codes were required. Codes were then reviewed and organized into three key domains: Bridging silos, Attitude change and Self-care. Quotes were selected to illustrate each of these domains.

## Results

Fifty-two workshops were held across the province of Ontario in both urban and rural settings. The number of participants attending a workshop ranged from 19-32 with an average of 23 participants. One planned workshop had fewer than 19 registrants in the week prior and was cancelled. Those individuals were encouraged to register for a workshop on another day in a nearby community. Workshops were facilitated by a Making Connections core facilitator and a local co-facilitator.

### Demographics

Participants (total *n* = 1111) were employed in VAW shelters (*n* = 262), worked as VAW counsellors (*n* = 237), in mental health (*n* = 229) or in addiction treatment settings (*n* = 167). A small number worked in “other” services/sectors (*n* = 149) while a few identified with more than one sector (*n* = 49); 18 left this unanswered. Over half of the participants (61%) worked in smaller organizations (<50 staff members). An almost equal number had between 6 and 15 years of experience as less than 5 years of experience (41% vs. 40%). More than half of the participants (63%) had some prior education on the intersections of domestic violence, mental health and substance use, while a little over one third reported no prior education (37%). Prior education was obtained through a variety of means including their workplace (37%), a conference (35%), or school (35%). More than one answer was possible. Nearly half (43%) stated their organizations did not have any policies, protocols or timeframes for supporting women with co-occurring problems (Table 1).

**Table 1:** Demographic characteristics of learners included in this study (*n* = 1111)

Characteristics	*N* (%)
Sector	
Violence Against Women – Shelter	262 (24%)
Violence Against Women	237 (21%)
Mental Health (not VAW)	229 (21%)
Substance Abuse/Addictions	167 (15%)
Other	149 (13%)
More than one sector	49 (4%)
Missing	18 (2%)
Age	
20–39 years	546 (49%)
40–59 years	468 (42%)
60+ years	70 (6%)
Missing	27 (2%)
Size of Organization	
Small (<50)	75 (61%)
Medium (<100)	172 (15%)
Large (>100)	213 (19%)
	41 (5%)
Years of Experience	
< 5 years	405 (36%)
6–15 years	456 (41%)
16+ years	225 (20%)
Missing	25 (2%)
Prior Education/Training on Intersection of DV, MH, and SU	
Yes, prior education acquired	670 (60%)
No	411 (37%)
Yes, during conference	388 (35%)
Yes, at workplace	412 (37%)
Yes, at school	397 (36%)
Yes, through self-study	261 (23%)
Yes, through other means	66 (6%)

### Pre/post-tests

Matched pre- and post-tests were available for just over half of the participants (56%). Across the six self-competency items, improvements from pre to post-test were significant (*p* <.0001) with greatest improvement shown in participants’ understanding of co-occurring problems (Table 2). Participants’ knowledge and stigmatizing beliefs improved from an average of 9.7 (pre-test) to 11.2 (post-test) with statistical significance (*p* <0.0001) using McNemar’s test. On all but three of the questions (6, 7, 11), statistically significant gains in knowledge and reductions in stigmatizing beliefs were achieved (Table 3). There were no significant correlations between participants’ test scores and age, sector of practice, size of organization, years of experience, or prior training.

**Table 2:** Learners’ self-rated competency scores from pre-test and post-test^a^

Competency Domain	*n*	Pre-Test		*n*	Post-test	
		Median (IQR)	Mean (SD)		Median (IQR)	Mean (SD)
Co-occurring Conditions^b^	617	3.00 (2.00–4.00)	3.0 (1.0)	620	4.00 (4.00–5.00)	4.2 (0.7)
Initiating Conversation^c^	618	3.00 (3.00–4.00)	3.3 (1.1)	620	4.00 (4.00–5.00)	4.3 (0.7)
Responding to Crisis^d^	616	3.00 (3.00–4.00)	3.2 (1.0)	619	4.00 (4.00–5.00)	4.1 (0.8)
Helping During Distress^e^	614	3.00 (2.00–3.00)	2.7 (1.1)	619	4.00 (3.00–4.00)	3.8 (0.9)
Building Organizational Partnerships^f^	615	3.00 (2.00–3.00)	2.5 (1.2)	619	4.00 (3.00–4.00)	3.8 (1.0)
Self-care^g^	618	4.00 (3.00–4.00)	3.5 (1.0)	620	4.00 (4.00–5.00)	4.3 (0.7)

^a^Sign test was used to compare the difference between the pre and post-test responses

^b^ “I understand the ways in which domestic violence, mental health, and substance use problems are interconnected.” ^c^ “I can initiate conversation, ask questions about, and appropriately refer a woman who has experienced domestic violence and has mental health and/or substance use problems.” ^d^ “I can respond to crises related to DV, mental health and/or substance use.” ^e^ “I can help a woman manage her distress even if she begins to dissociate while talking to me.” ^f^ “I can outline the steps to building useful organizational partnerships.” ^g^ “I can recognize the signs of burnout or compassion fatigue and have strategies for self-care

**Table 3:** Correct responses between pre and post-test (knowledge and belief based questions)^a^

Question No.	Pre-test (*n* = 624)	Post-test (*n* = 624)	*P*-value^b^
1	363 (58%)	500 (80%)	<0.0001
2	513 (82%)	567 (91%)	<0.0001
3	314(50%)	439 (70%)	<0.0001
4	228 (37%)	419 (67%)	<0.0001
5	540 (87%)	573 (92%)	0.0007
6	572 (92%)	558 (89%)	0.09
7	599 (96%)	602 (96%)	0.61
8	512 (82%)	551 (88%)	0.0004
9	367 (59%)	464 (74%)	<0.0001
10	537 (86%)	570 (91%)	0.0005
11	234 (38%)	255 (41%)	0.18
12	513 (82%)	573 (92%)	<0.0001
13	243 (39%)	315 (50%)	<0.0001
14	548 (88%)	595 (95%)	<0.0001
Total Correct Answers^c^	9.7	11.2	<0.0001

^a^Unanswered questions were considered incorrect

^b^McNemar’s Test was used to compare the difference between pre and post-test responses

^c^For average correct answers, a paired *t* test was used

### Workshop evaluations

Workshop evaluations were completed by 86% of participants and were almost uniformly positive in their overall assessment of the value of the training. In response to the open-ended question, “tell us one thing that you have learned today and will use,” comments clustered into the importance of bridging practice silos to holistically address clients’ needs (41%), having stigmatizing attitudes and practices challenged (24%), and the reminder of the need for self-care (9%).

#### Bridging Silos

Included within this domain were codes related to connections, networking, collaboration, community building, community contacts, and new resources.

Learning about and connecting with individuals from other services and sectors was the most frequently cited benefit of the workshop. Participants recognized that many of their clients had experiences in common irrespective of the service accessed (i.e. mental health, addiction treatment, or VAW shelter or counselling). Participants were excited about meeting individuals from other sectors with whom they could consult when needed.

“I often will focus on the abuse and mental health issues that surround my clients and not explore the addiction side. I will now look into how all three of them go hand in hand.”

“[I learned] About other community agencies and services and their referral processes. Will be helpful when working with clients to know how to help address more of their life challenges in addition to their addiction.”

“[I learned] How important it is to acknowledge that issues run concurrently and to not only treat them individually.”

Almost equally important to participants was the opportunity to learn more about the co-occurring problems they were seeing in their clients. Many commented that because of what they had learned, they would be better able to help their clients at the point of contact.

#### Attitude Change

Included within this domain were codes related to: stigmatizing beliefs, challenging beliefs, better understanding, client as expert, thinking before speaking, asking questions, and choosing words wisely.

A number of participants reported having their own misconceptions about women with co-occurring problems challenged while others acknowledged the impact that stigmatizing beliefs had on their own practices. Also highly valued was learning alternative approaches to asking clients about their experiences.

“Understanding that mental health issues are often issues that arise from experiencing domestic violence, and [I learned that] it is important to be open and non-judgmental as you never know what each person has experienced.”

“[I learned]…The value of working from a strength based perspective. A woman wants to be viewed as a whole person not just as her issues and problems.”

#### Self-care

Included within this domain were codes related to well-being, loving oneself, putting oneself first, and guilt.

The need for frontline workers to recognize their own limitations and maintain their own well-being was the focus of the workshop’s final session. Although we initially thought this session would be the least valued activity, in general the comments were extremely positive and the session was greatly appreciated.

“I expected the self-care component to be “Oh yeah, I already know that” but I was surprised at how much I learned and need to apply. I’m excited to be able to share this with some of my friends back at my old workplace who just emailed me an SOS. Thanks for that!”

## Discussion

Women who experience co-occurring IPV and mental health and/or substance use problems have often found themselves shunted from service to service when seeking help for problems that are inextricably connected. Funding mechanisms, mandates, philosophies, language, values, and jurisdictional issues have led to service delivery silos and institutionalized barriers. Upon accessing care, survivors may then encounter providers who consciously or unconsciously harbor common myths and ‘victim-blaming’ attitudes which can negatively impact recovery [21–23]. In developing an intervention to sensitize workers to the realities of women’s experiences of co-occurring problems, educate about the challenges of accessing help when there are co-occurring problems, and bridge discipline and practice-based silos, we hoped to break down silos allowing providers from different sectors to share perspectives, develop appreciation for each other’s expertise, and challenge and alter any negative attitudes or stigmatizing beliefs. Developing personal relationships with individuals from other sectors will support the process of referring clients to appropriate services – and perhaps even lead to more and better integrated care. Decreasing stigmatizing beliefs and increasing knowledge has the potential to increase appropriate responses from healthcare and other providers, thereby helping survivors access the help they need [24–26].

Similar to other studies, we have shown that a well-designed educational intervention can effectively change providers’ stigmatizing beliefs and increase their knowledge about IPV [27, 28]. Unlike previous studies, we have also demonstrated that a significant component of the curriculum can be delivered through an accessible online curriculum, potentially reaching more providers than would be possible through workshops or classroom teaching. Although the in-person workshops are no longer supported through our offices, to date an additional 1,239 individuals have accessed the online modules alone, meaning the total number of curriculum participants is now 2,350. The online modules and text manual were designed to promote inter-professional/cross-disciplinary education and enhance mutual understanding; the fact that they will endure beyond the time limits imposed by funding is very gratifying. The in-person workshop supported these objectives but additionally demonstrated the importance of creating opportunities for local providers to develop greater familiarity with local resources and begin the work of building new networks and collaborations. Finding alternative ways of bringing individuals from different sectors together remains important as IPV survivors who also experience substance use or mental health problems suffer from the lack of coordinated services [29] which increases the likelihood of service failure, future injury, and relapse for women who have experienced violence [30]. Improved health and wellbeing occur when care is coordinated and services streamlined [31].

### Limitations

This study has some important limitations. Similar to other interventions [28], the post-test response rate was quite low (55%) which may have led to skewed results. Post-tests were sent three months after completion of the program and up to four reminders were sent out monthly, meaning some respondents completed at three months, while others responded four or five months post-workshop. This too may have affected scores although one would assume knowledge would have been lost over time resulting in lower scores, which does not seem to have been the case. Also our participants were largely self-selected suggesting they had a prior interest in learning about co-occurring problems for women experiencing IPV and this may limit the generalizability of our findings.

## Conclusion

This study offers support for a multi-modal educational intervention on IPV, substance use and mental health to improve frontline workers knowledge, beliefs and skills in helping the women who experience these problems. Given the need for evidence informed interventions to address these comorbidities, this training intervention has the potential to benefit frontline workers and may improve outcomes for women with co-occurring issues by ensuring they get supportive, coordinated, accessible care. Inter-professional approaches to education and training on IPV, mental health and substance use are effective and should be implemented in order that care providers are able to deliver appropriate support to the women who experience these problems.

## Additional file

Additional file 1: Pre-test knowledge and beliefs about co-occuring IPV, mental health, and substance use. Description of data: Multiple choice pre-test given to all participants (DOCX 14 kb).

## Abbreviations

IPV: Intimate partner violence
IQR: Interquartile range
SAMHSA: Substance abuse and mental health services administration
VAW: Violence against women
