# Supplementary material for: Making connections across silos: intimate partner violence, mental health, and substance use
Source: BMC Womens Health. 2017 Apr 12;17:29. doi: 10.1186/s12905-017-0372-4 (PMC5389007; doi:10.1186/s12905-017-0372-4)
Supplement: Additional file 1: — Pre-test knowledge and beliefs about co-occuring IPV, mental health, and substance use. Description of data: Multiple choice pre-test given to all participants (DOCX 14 kb). [file 12905_2017_372_MOESM1_ESM.docx]

**Questionnaire of pre-test knowledge and beliefs**

1. Which of the following is generally **not** considered a determinant of health?
   1. Income and social status
   2. Gender
   3. Culture
   4. Religion
   5. Social support networks
   6. Education and literacy
2. Legal substances, specifically alcohol and tobacco, cause more harm to more women than do illegal substances.
   1. True
   2. False
3. ________ of Canadian women over age 16 have experienced at least one incident of physical or sexual violence.
   1. 5%
   2. 25%
   3. 50%
   4. 75%
4. According to Ontario’s Domestic Violence Death Review Committee, which of the following is **not** a common risk factor for lethal violence:
   1. Victim’s intuitive sense of fear
   2. Perpetrator depressed in professionals’ (e.g. physician, counsellor) and/or non-professionals’ (e.g. family, friend) opinion
   3. Actual or pending separation
   4. Perpetrator’s broken promise to seek help
   5. Prior threats/attempts to commit suicide
   6. History of domestic violence
5. How many Canadians will personally experience a mental health problem at some point during their life?
   1. 1 in 5
   2. 1 in 25
   3. 1 in 50
   4. 1 in 100
6. Domestic violence always precedes mental health or substance use problems.
   1. True
   2. False
7. Mental health or substance use problems may increase the risk of domestic violence.
   1. True
   2. False
8. Which of the following is **not** a factor in considering the immediate safety of a woman experiencing DV? (check all that apply)
   1. The pattern and history of abuse
   2. The level of immediate risk and potential lethality
   3. The family’s social class or status
   4. The presence of children in the home
   5. Her level of isolation or support
9. The most social criticism and stigma is directed at:
   1. Young pregnant women
   2. Poor women with mental health problems
   3. Pregnant substance using women
   4. Women who spank their children in public
10. Pregnant women who are heavy substance users should be encouraged to immediately stop even if they have experienced serious withdrawal symptoms in the past.
    1. True
    2. False
11. In the context of mental health, a “trigger” refers to (check all that apply):
    1. A reminder of past danger
    2. Activation of the individual’s alarm system
    3. Part of the brain’s persistent defensive reaction system
    4. An example of the “fight” response to stress or danger
12. Which of the following is **not** a good strategy to use when helping a woman manage signs of dissociation?
    1. Active listening
    2. Grounding
    3. Talking about the trauma
    4. Breathing exercise
13. Working with women who have experienced DV, mental health and/or substance use problems can result in (check all that apply):
    1. Burnout
    2. Systemic disorder
    3. Secondary traumatic stress
    4. Compassion satisfaction
14. Which of the following is not part of the Stage of Change Model?
    1. Pre-contemplation
    2. Testing and fine tuning
    3. Action
    4. Maintenance
